# Immune Monitoring in Cancer Vaccine Clinical Trials: Critical Issues of Functional Flow Cytometry-Based Assays

**DOI:** 10.1155/2013/726239

**Published:** 2013-09-30

**Authors:** Iole Macchia, Francesca Urbani, Enrico Proietti

**Affiliations:** Department of Hematology, Oncology and Molecular Medicine, Istituto Superiore di Sanità, 00161 Rome, Italy

## Abstract

The development of immune monitoring assays is essential to determine the immune responses against tumor-specific antigens (TSAs) and tumor-associated antigens (TAAs) and their possible correlation with clinical outcome in cancer patients receiving immunotherapies. Despite the wide range of techniques used, to date these assays have not shown consistent results among clinical trials and failed to define surrogate markers of clinical efficacy to antitumor vaccines. Multiparameter flow cytometry- (FCM-) based assays combining different phenotypic and functional markers have been developed in the past decade for informative and longitudinal analysis of polyfunctional T-cells. These technologies were designed to address the complexity and functional heterogeneity of cancer biology and cellular immunity and to define biomarkers predicting clinical response to anticancer treatment. So far, there is still a lack of standardization of some of these immunological tests. The aim of this review is to overview the latest technologies for immune monitoring and to highlight critical steps involved in some of the FCM-based cellular immune assays. In particular, our laboratory is focused on melanoma vaccine research and thus our main goal was the validation of a functional multiparameter test (FMT) combining different functional and lineage markers to be applied in clinical trials involving patients with melanoma.

## 1. Introduction

The primary objective of immune monitoring in cancer vaccine clinical trials is to find a correlation between the efficient induction of tumor-specific T-cell responses and clinical efficacy, due to the importance of the host immune system in controlling tumor progression. However, although in several cancer vaccine trials there is indication of increased frequency of tumor-specific T-cells, no validated biomarkers exist for cancer immunotherapy as yet [1].

One reason for the lack of correlation between the immune parameters measured and objective clinical response might be the complexity of the immune responses required for a successful tumor eradication which cannot be dissected through the most frequently used T-cell assays.

Antitumor cell-mediated immunity is a key biomarker for most vaccines and immunotherapies and involves the activity of specialized cells including antigen specific cytotoxic T lymphocytes (CTLs) [2] and CD4^+^ helper T lymphocytes [3] as well as components of innate immunity such as macrophages, dendritic cells (DC), natural killer cells (NK), and granulocytes.

In addition, since the success of immune response against cancer is due to the balance between the effector and the suppressive compartments, immunological monitoring should also include analysis of immune suppressive cells (ISCs) such as regulatory T-cells (TREGs), myeloid-derived suppressor cells (MDSCs), and tumor-associated macrophages (TAMs) [4–6]. These cells play an important role in cancer progression and responses to immunotherapy. However, due to their tremendous phenotypic and functional heterogeneity, their usefulness as biomarkers of outcome or response to therapy has to await for further development of monitoring assays that better reflect their biologic significance in cancer.

In addition, a recent report [7] indicates that regulatory B cells (BREGs), a small subset of suppressor cells, may have profound effects on the development of T-cell responses, further complicating the interpretation of antitumor immune suppression in disease.

Local and systemic antitumor immune responses can show markedly different patterns and many functional responses could be missed when only peripheral blood lymphocytes (PBLs) and not tumor infiltrating lymphocytes (TILs) are evaluated; therefore, more emphasis should be put on immune monitoring also at the effector site by taking a biopsy of a metastatic lesion [8]. To this end, the concept of “Immunoscore,” initially described for colorectal cancer patients, has been recently introduced as an essential prognostic and potentially predictive tool to classify cancers, beside the traditional tumor staging classification (AJCC/UICC-TNM) [9–13]. This parameter might facilitate clinical decision making including rational stratification of patient treatment. Usually the immunoscore approach refers to the analysis of the location, density, and functional orientation of different immune cell populations infiltrating the tumor, including macrophages, DC, mast cells, NK cells, naïve and memory lymphocytes, B cells, and T lymphocytes (which include various subsets of T-cell: T_H_1, T_H_2, T_H_17, regulatory T-cells (T_REGS_), T follicular helper cells (T_FH_), and cytotoxic T-cells). Such “immune contexture” annotated in a large collections of human tumors has allowed the identification of components that are beneficial for patients and those that are deleterious [14]. For instance, in a study by Pagès et al. [15], high densities of T-cells (CD3^+^), cytotoxic T-cells (CD8^+^), and of memory T-cells (CD45RO^+^) were clearly associated with a longer disease-free survival (DFS) (after surgical resection of the primary tumour) and/or overall survival (OS).

However, the analysis of tumor microenvironment is not always feasible and the only samples available are those obtained from peripheral blood. Furthermore, sometimes peripheral responses should be of some relevance and could integrate and increase information given by the tumor microenvironment. For this reason, we believe that, in addition to scoring T-cells at tumor sites, the frequency and functions of T-cells circulating in the peripheral blood of cancer patients should be examined as potential biomarkers, by means of validated and standardized immune assays.

Concerning the quality of T-cell response, several papers showed that the multifunctionality of effector cells is an important factor to predict the immunological protection [25]. In particular, it has been demonstrated that the functional profile of HIV-specific CD8^+^ T-cells in progressors is limited compared to that of nonprogressors, which consistently maintain highly functional CD8^+^ T-cells and that the frequency and proportion of the HIV-specific T-cell response with highest functionality inversely correlates with viral load in the progressors [26]. In addition, other reports indicated that vaccine-induced multifunctional CD4^+^ and CD8^+^ T-cells produce greater amounts of IFN-*γ* than cells that secrete IFN-*γ* alone [27].

In the setting of cancer immunotherapy, the induction of polyfunctional NY-ESO-1-specific T-cell responses, following anti-CTLA-4 treatment of metastatic melanoma patients, has been recently shown to enhance T-cell responses and to induce durable clinical responses [28]. Further, a recent paper [29] demonstrated that the triple combination of IFN-*γ*, IL-2, and TNF-*α* represents a Th1 pattern of polycytokine secretion with greater antigen sensitivity and superior tumor recognition.

In order to get new insights in exploitation of vaccine-induced polyfunctional T-cells, standardization and validation of multiparameter assays are required.

In this review, we will overview the current technology used for immune monitoring during cancer immunotherapy in melanoma patients, focusing on a polychromatic FCM-based approach for *ex vivo* detection of tumor-antigen specific T lymphocytes producing multiple functional molecules simultaneously. To this aim, we will provide few experimental examples to discuss critical process steps encountered during validation of an FMT developed in our laboratory, consisting of a six-color panel for assessment of polyfunctionality of tumor-specific CD8^+^ T-cells in cryopreserved human peripheral blood mononuclear cells (PBMCs).

## 2. Overview of Immunoassays

The objective of any immune monitoring study is to collect interpretable, reliable, and reproducible data for the detection, quantification, and characterization of immune responses directed at specific antigens.

The principal techniques utilized for immune monitoring are reviewed in [30].

Measuring cytokine production and profile represents an integral part of immune monitoring during immunotherapeutic treatments [31]. First-generation immune-monitoring techniques included proliferation and cytotoxicity assays following short-term *in vitro* expansion; more recently, tetramer and Elispot (second-generation assays) allowed to assess directly *ex vivo* the frequency of vaccine specific T-cells and their ability to produce cytokines, usually IFN-*γ* [32]. However, this type of analyses is limited by the lack of information about the functional state of the cells. In the setting of cancer patients, where tumor escape mechanisms may induce T-cell anergy by altering lymphocyte signalling and effector functions [33], the need for third-generation assays aimed at evaluating the functional properties of rare cell populations of vaccine-induced T-cells with a multiparameter approach is becoming increasingly evident. To this aim, the development of polychromatic flow cytometry for immune monitoring has significantly contributed to progress in the field of human immunology.

In this paper, we will focus on some of the most widely used FCM-based assays for measurement of antigen-specific T-cells. In particular, intracellular cytokine staining (ICS) represents one of the main FCM-based assays and it has been previously validated by Horton et al. and De Rosa et al. [24, 34].

The CD107 mobilization assay measures the exposure of CD107 (LAMP: lysosomal associated membrane protein) a and b, present in the membrane of cytotoxic granules of CTLs, onto the cell surface as a result of degranulation and it can be used as an alternative to ^51^Cr release assay. In fact, a good correlation has been demonstrated between degranulation and cytotoxic activity of tumor-specific CD8^+^ T-cell clones and CD8^+^ T-cells, as measured in an FCM-based killing assay [35, 36]. Further, CD107-expressing CD8^+^ T-cells are shown to mediate cytolytic activity in an antigen-specific manner. Soluble major histocompatibility class I tetramers are a widely utilized tool for the direct *ex vivo* detection, characterization, and isolation of antigen-specific T-cells in a variety of clinical settings such as infectious, autoimmune, or neoplastic diseases [37–41].

To provide a more complete assessment of the functionality of CD8^+^ T-cells expressing cognate T-cell receptors (TCR), measurement of CD107a and b expression can be combined with MHC-class I tetramer labeling and ICS [35].

In order to insure reproducibility and worldwide comparisons for conclusive longitudinal monitoring in multicenter studies, standardized operating procedure (SOPs), as well as standardized reagents and analysis protocols, need to be used [42].

Effective large-scale assay harmonization efforts have already been conducted for commonly used immunological assays of peripheral blood immune cell populations [43, 44].

Advances in multiparameter flow cytometric technologies and reagent applications for characterization and functional analysis of cells modulating the immune network have been recently reviewed in [45].

Researchers from Europe and the United States have started a project called Minimal Information About T-cell Assays (MIATA) [46] to standardize and harmonize commonly used assays such as the enzyme linked immunosorbent spot assay (Elispot) [47] and major histocompatibility complex tetramer assays [48]. The establishment of universally accepted guidelines for performing and presenting immunological assays in scientific publications will create a framework that will allow the comparison of immune responses across clinical trials. Many groups performed also optimization and harmonization of intracellular cytokine assays [49–52].

## 3. Key Issues Involved in FCM-Based Assays and Development of FMT

Flow cytometry is a powerful and versatile technique, ideal for phenotyping, enumerating, and assessing the function of rare and precisely defined cell subsets at the single cell level. Functions assessed by flow cytometry include cytokine/chemokine production, CD107 expression, multimer analysis, natural cytotoxicity, antibody dependent cell cytotoxicity (ADCC), and proliferation. Critical steps for immune monitoring by flow cytometry which may affect yield, viability, and immunologic function of cells, include shipping blood variables such as temperature [16] and time delay of drawn blood processing, freezing/thawing conditions [17, 19, 51], type of anticoagulant used for blood collection, and type of density-gradient centrifugation used for the isolation of peripheral blood mononuclear cells. Other variables facing multicolor assay depend on antibodies and fluorochromes used, fixation and permeabilization reagents, instrument setup, data acquisition and analysis, reporting of results, internal quality control, external quality assessment, and flow sorting [53] (Table 1).

The importance of *ex vivo* analysis versus *in vitro* analysis has been addressed by [54].

The principal challenge for FCM-based assays for immune monitoring in cancer clinical trials is often due to the need of detecting rare subsets of cells avoiding the spurious positive events. This goal can be achieved by using a multiparameter approach in order to minimize the false positive and negative events by gating and subgating the cells of interest which express multiple markers simultaneously. Other critical issues are represented by *in vitro* T-cell culture methods when immune responses are analyzed in expanded T-cell cultures instead of *ex vivo*. To this aim, optimization of a cell culture method for analysis of polyfunctional T-cells has been previously dissected [55].

By setting up a procedure to assess polyfunctionality of tumor-associated antigen- (TAA-)specific cells in clinical trials, we observed a reduction of cell number at the end of the experiment, probably due to a loss of cells at different steps of cell processing (unpublished data). These observations led us to initiate a set of controlled *in vitro* studies to investigate the impact of different reagents and methods on recovery, viability, and immunological function of cells.

Our final goal was the optimization and validation of a reliable method, which we named FMT, for assessment of antigen specificity and effector functions against the melanocyte differentiation antigen Melan-A/MART-1. This protocol, adapted from [56], was based on a six-color panel combining CD8, MHC-tetramer, CD107a, and intracellular cytokine staining for three soluble factors with distinct properties (CD107, TNF-*α*, and IL-2), in response to the peptide Melan-A/MART-1 or other stimuli.

**Figure 1:**
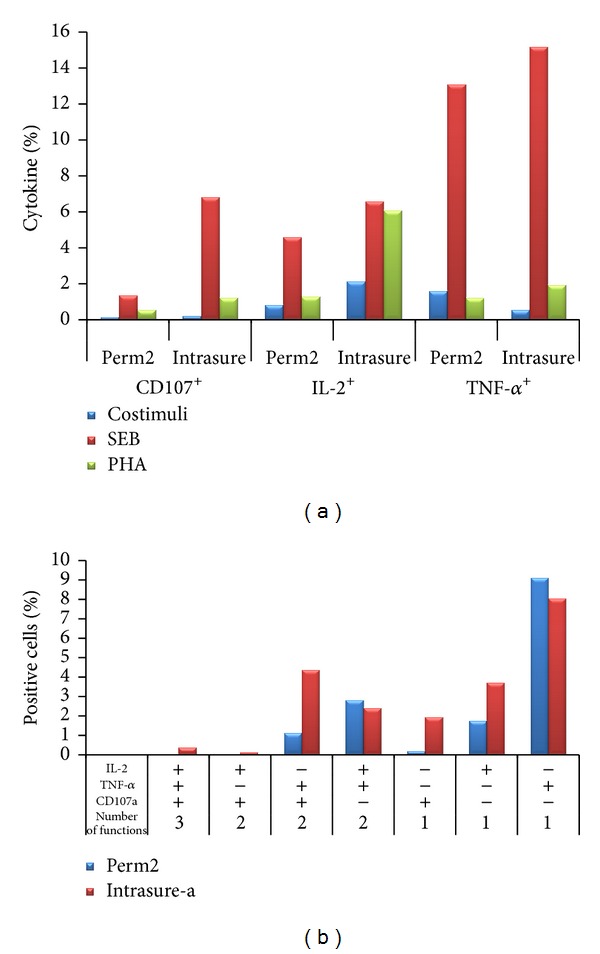
Comparison of intracellular and cell surface markers after treatment of cells with two different fixation/permeabilization buffers. Assessment of cytokine secretion and cytotoxic factor expression in CD8^+^ T-cells. Briefly, thawed PBMC from a healthy donor was cultured (1 hour at 37°C) in presence of anti-CD107a and *Staphylococcus* enterotoxin B (SEB; Sigma-Aldrich, Munich, Germany, used at 2 *μ*g/mL) or PHA (HA16, Murex Biotech, Dartford, UK, used at 1.5 *μ*g/mL) in presence of costimulatory antibodies (CD28 and CD49d). After the addition of brefeldin A (Golgi Plug) and monensin (Golgi stop) (Becton Dickinson, San Jose, CA, USA), cells were incubated for additional 5 hours. Following stimulation, final 2 mM EDTA was added to each well and incubated for 15 minutes. Cells were then incubated for 30 min at 4°C with surface antibodies (CD8), fixed, and permeabilized with the previously mentioned lysing/permeabilization buffers and stained with fluorescently labelled antibodies directed against IL-2 and TNF-*α*. Samples were then acquired on a FacsCanto flow cytometer instrument (BD Biosciences) and analyzed by FACSDiva and/or FlowJo software (Tree Star, Ashland). (a) Bar graph showing the percentages of total CD107a^+^, TNF-*α* ^+^, and IL-2^+^ analyzed within CD8^+^ gated cells. (b) Bar graph showing the polyfunctionality of CD8^+^ T-cells upon SEB stimulation (Boolean analysis). As negative controls, we included untreated cell (only costimuli).

In previous experiments, based on previous reports indicating that different fixation/permeabilization buffers may affect the results of intracellular cytokine detection [21], we performed the FMT for CD107, TNF-*α*, and IL-2, after stimulation with *Staphylococcus* Enterotoxin B (SEB) and PHA and we compared two distinct standardized commercial lysing/permeabilization buffers: the Lysing/Perm solutions and the Intrasure kit (both purchased from BD Biosciences) (Figure 1).

Overall, we found that the fixation/permeabilization with the Intrasure kit resulted in a stronger response for all the parameters analyzed and that stimulation with SEB yielded the higher percentages of CD8^+^ T-cells producing one, two, or three factors (Figure 1). Based on these results, we decided to use in our next experiments the Intrasure permeabilization kit and SEB as positive control.

Next, we investigated the impact of DNAse, which is usually used to digest extracellular DNA and reduce cellular clumping, on cell recovery and viability as well as its effect on cell function (Table 2). 

Compared to previous reports [20] facing this issue, we try to keep the DNAse during the all steps of the FMT procedure, from thawing to culturing, even during washing steps.

Our results indicated that using DNAse after thawing PBMCs samples and during the entire procedure increased the absolute number and percentages of CD8^+^/MART-1+ cells as shown in Table 2 that summarizes the effect of DNAse on cell recovery at the end of FMT, by enumerating TAA-specific (Melan-A/MART-1) CD8^+^ T-cells in the presence or not of DNAse (DN25- SIGMA).

Soluble tetrameric MHC/peptide complexes have opened the possibility to directly identify and monitor antigen-specific CD8^+^ T-cells at the tumor site and in blood [40]. Multiparameter monitoring of antigen-specific T-cell responses that combines *ex vivo* tetramer staining with various phenotyping and functional assays provides a novel approach to assess the functional potential of tumor-specific T lymphocytes and may also facilitate the optimization of vaccination protocols.

Dextramers are multimers based on a dextran backbone bearing multiple fluorescein and streptavidin moieties, used for the analysis of relatively low frequency antigen-specific T-cells in peripheral blood. The functionality and optimization of dextramers have been previously demonstrated on human CD8^+^ T-cell clones with four independent antigen specificities [57].

**Figure 2:**
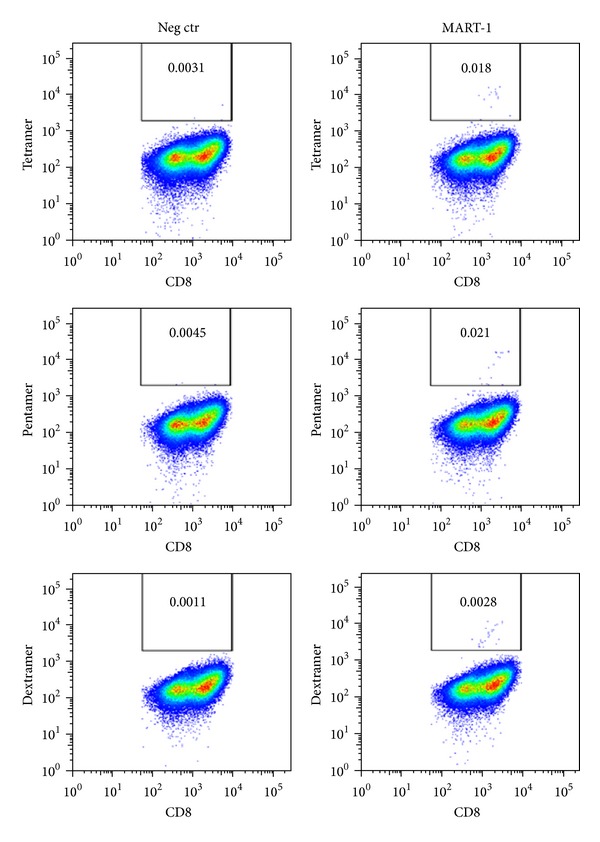
Comparison of different MHC multimers for detection of antigen-specific T-cells: dot plots representing percentages of CD8^+^ MART-1^+^ tetramer^+^/pentamer^+^/dextramers^+^ cells, analyzed within the singlets-live gate of a CD8^+^ expanded line obtained from a healthy donor.

Staining of a CD8^+^ line from a healthy donor with either MART-1-specific tetramers or pentamers or dextramers shows that dextramers produce a stronger signal against Melan-A antigen and a lower background signal than their tetramer and pentamer counterparts (Figure 2). Thus, dextramers could become the reagents of choice as the antigen-specific T-cell labeling transitions from basic research to clinical application.

Finally, validation of the FMT for analysis of the functionality of T-cells directly *ex vivo* was performed on a melanoma patient with discrete percentage of CD8^+^ MART-1^+^ specific T-cells.

**Figure 3:**
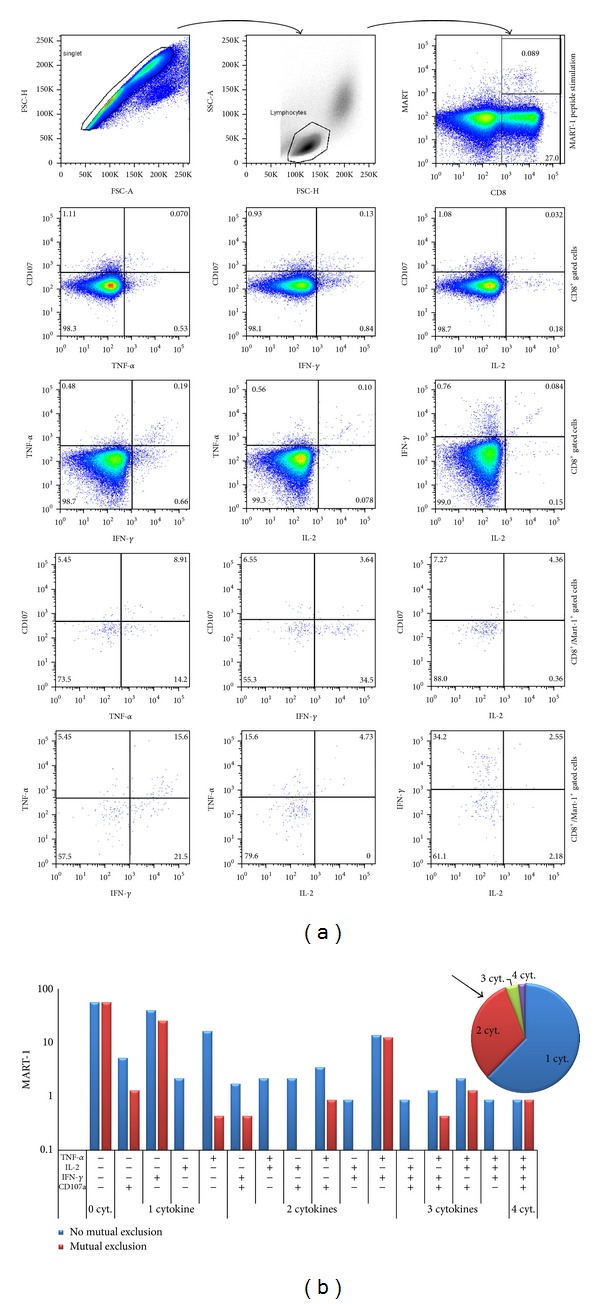
Validation of FMT (representative example of a melanoma patient). (a) Sequential gating strategy. Representative example dot plots and FACSDIVA analysis. Forward-scatter (FSC) area versus FSC height parameters were used to exclude cell doublets; cells were gated by forward and side scatter for lymphocytes; gated populations are plotted as CD8 (horizontal axis) versus tetramer staining (vertical axis). Direct *ex vivo* analysis cytokine production (IFN*α*, TNF*γ*, IL-2) and degranulation CD107a/LAMP-1) within CD8^+^ population or CD8^+^/MART-1 tetramer gated T-cells after stimulation with Melan-A/MART-1 peptide. (b) Histogram plots representing the individual functional combinations as a proportion of the total responding cells after stimulation with Melan-A/MART-1 peptide. *Mutual exclusion, red bars*: Percentage of cells expressing a certain combination of parameters (+) and not expressing the parameter indicated as (−). *No mutual exclusion, blue bars*: percentage of cells expressing a certain combination of parameters (+) independently from the expression of the parameter indicated with (−). The *pie slices* indicate the average proportion of the response producing 1, 2, 3, or 4 functions (regarding in this case “no mutual exclusion” variables). Each slice indicates one of the functions.

In this assay, we evaluated the production of multiple cytokines (IFN*α*, TNF*γ*, and IL-2) and upregulation of LAMP-1 (CD107a) by tumor- (Melan-A/MART-1) specific T-cells. (Figures 3(a) and 3(b)).

On our side, our FMT experiments were acquired on a BD-Canto instrument by DIVA software. We chose to analyze them by a classical approach, using a standard software dedicated to flow cytometry analysis, Flow Jo (Treestar, MA, USA) and generating graphical representation by Excel (Microsoft, WA, USA) elaboration. Gating strategy might have a potential impact on the analysis of antigen-specific polyfunctional T-cell responses. In our setting, our population of interest was defined meeting the criteria of a lymphocyte morphology, based on forward- and side-scatter parameters, singlet morphology, based on forward height scatter and forward area scatter, positivity of surface antigen expression (tetramer and CD8). Expression of 4 parameters, CD107a and the intracellular cytokines, IL-2, IFN-*γ*, and TNF-*α*, was simultaneously investigated by a 6-colors staining to assess polyfunctionality of the gated population. This sequential gating strategy is shown in Figure 3(a), along with some bidimensional plots showing some of the possible representation of parameters under study.

The possible combinations of positivity/negativity of these 4 parameters generate a large number of variables (30 for each sample). An effective way to give a graphic representation of such a lot of variables is to use histograms and pie chart (Figure 3(b)). Pie charts give a quick shot of the proportion of responding cells producing one or more functions without specifying which is the particular function [58]. We drew these graphics by elaborating FlowJo results export using a standard Excel (Microsoft, WA, USA) worksheet, adapting the Simplified Presentation of Incredibly Complex Evaluations software's approach (SPICE, Version 2.9, Mario Roederer, Vaccine Research Center, NIAID, NIH), one of the most largely utilized free Apple Mac-based data mining software [54, 58, 59].

## 4. Tools and Software for Analysis of Flow Cytometric Data

Traditional methods to analyze flow cytometric data involve gating of populations in one- or two-dimensional displays and manually selecting populations of interest. However, such methods are highly subjective and time consuming. Particularly, with the advent of multiple lasers flow cytometry analyzer, it is possible to have up to 18 colors of fluorescence detection simultaneously in the same sample. This leads to an enormous amount of variables, due to the all possible combinations of each parameter acquired. So that, critical is the analysis approach: bioinformatics will be surely the way to manage this kind of data in the next future. Looking back to the classical way to analyze FCS files, by manual, sequential gating, in the past years an enormous number of dedicated software has been developed by industries and academies (most of these last being freeware) [60].

Just to cite the most common among them, BD-DIVA, Miltenyi-MACSQuantify, Millipore-GuavaSuite, (acquisition and analysis commercial software, being interfaces of flow cytometer), FlowJo (one of the most common analyser software for flow cytometry), BeckmanCoulter-Kaluza, Weasel (commercial analysis software), and WinMDI, (free academic analysis software); each of them is endowed with peculiar tools and utility.

A detailed list of cytometry software and educational materials in cytometry is provided by the “original cytometry software catalog,” developed and managed by Dr. Eric Martz and by the Purdue University Cytometry Laboratories (http://www.cyto.purdue.edu/flowcyt/software/Catalog.htm).

Reviewing the new computational approach of analysis, often based on automated gating and high level of statistical analysis and representation output, several groups have developed different strategies.

Among them, open source tools like Bioconductor flowFlowJo, able to extracting information from a FlowJo workspace and deliver the data into *R* (one of the most common statistical processor) in the flowCore paradigm, have been developed to allow the management of high throughput data [61].

Probability binning algorithm extensively described by Roederer et al. in [62] is today a powerful tool employed, that is, to define subpopulation of cells, by means of-high resolution phenotype [63].

One unique approach, an algorithm called SPADE, utilizes downsampling, clustering, minimum spanning tree, and upsampling algorithms to generate two-dimensional branched visualizations [64, 65]. The branched tree structure incorporates information from all measurements in the data, partially addressing scalability issues. However, SPADE has many of the same subjective inputs as conventional clustering algorithms (e.g., number of clusters) and also may have issues of reproducibility and generation of nonbiological branches. 

Similar to the SPADE software, the Euroflow Consortium software called INFINICYT uses nearest-neighbor analysis to associate the data around the center of the mass of cells. Adopting Euclidean distance analysis, this software associates a normal profile for a cell type (through phenotyping of multiple normal samples) to identify and characterize an abnormal profile (http://www.infinicyt.com/). Developed as a diagnostic tool, this approach is limited by the relative frequency of the cell subset of interest and restriction of the parameter chosen to determine the normal profile that was used to create the database.

An additional way to look at the data is using the probability state modeling (PSM) method and the visualization tools in GemStone (http://www.vsh.com/products/gemstone/) software for the analysis of multidimensional flow cytometry data. A probability state model is a set of generalized *Q* functions, one for each correlated measurement, where the common cumulative probability axis can be a surrogate for time or cellular progression. By exploiting the unique characteristics of *Q* functions, PSM can model any number of correlated measurements and present one comprehensive yet understandable view of the results. In summary, these various software packages work to reduce the complexity into a relatively small set of model parameters that are amenable to group statistics and comparisons.

A model-based analysis based on statistical mixture models has been recently reported by [66], for cell subtype identification in flow cytometry.

Multivariate analysis of flow cytometric data using decision trees is interestingly described in [67], where, in order to examine whether the production of a certain cytokine is depended on other cytokines, datasets from intracellular staining for six cytokines with complex patterns of co-expression were analyzed.

A number of these approaches involve some variation of clustering analysis, which can have important limitations. Other approaches have been developed in addition to clustering, including principal components analysis (PCA) and Bayesian inference. These approaches have been evaluated through the FlowCAP initiative (http://flowcap.flowsite.org/).

To standardize the *universe* of Flow Data, MIFlowCyt (minimal information about a flow cytometry) experiment standard has been approved by the International Society for Advancement of Cytometry for the reporting of any flow cytometry results (http://flowcyt.sourceforge.net/miflowcyt/).

Our comment on computational approach, using of command line languages, algorithm design is that it is a potent and precious tool but quite far from the mean expertise of a flow cytometer user, which should have a specific training and/or collaborating with a bioinformatician. Maybe the future will reserve us more user friendly interfaces dedicated to computational analysis of flow data.

## 5. Consortia and Useful Links for Harmonization and Standardization of FCM-Based Assays

Many immune monitoring Consortia have been recently created worldwide to help facilitate and harmonize immune monitoring approaches in the cancer immunotherapy field and establish rigorous quality control standards for serial monitoring of immunologic functions.

Among them, the Cancer Immunotherapy Consortium (CIC) of the Cancer Research Institute (CRI) in USA and the Association for Cancer Immunotherapy (CIMT) in Europe supported the web-based reporting framework on “Minimal Information about T-Cell Assays” MIATA [46], a project aimed at generating recommendations on the minimum information required to allow an objective and thorough interpretation of published results from immunological T-cell assays. (http://www.miataproject.org/). As mentioned before, up to date, this framework has completed proficiency panels and published harmonization guidelines for the top immune assays (Elispot, peptide-multimer assays, intracellular cytokine staining, and Luminex) [48, 68].

In addition, several links might help researcher to find their response to common questions regarding technical issues about FCM based-assays. Just to cite some of them, the Maecker lab weblog (http://www.miataproject.org/) provides research and training materials for flow cytometry and immune monitoring; the (http://cytobank.org/facselect/) might help to assist with optimization of staining conditions; the ICH Q2(R1) document (http://www.ich.org/products/guidelines/quality/article/quality-guidelines.html) is a guideline for validation of analytical procedures. In addition, a number of companies, such as the IST (http://immunositetechnologies.com/services/automation/immune-monitoring-automation.html) offer their expertise to design, develop, validate, and run polychromatic flow assays.

## 6. Conclusions

The development of new vaccines and immunotherapeutic strategies against cancer requires the sophisticated assessment of immune parameters (biomarkers) to reliably measure antitumor immune responses.

A wide range of advanced monitoring assays is currently used to determine phenotypical and functional characteristics of antitumor T-cells in cancer immunotherapy trials, including T-cell proliferation, cytokine profile, CTL assays, CTL-associated molecules (CD107, perforin, granzyme B, and CD154), and MHC-multimer analysis. However, these assays still fail to establish the possible correlation between immune response and clinical outcome. The lack of this correlation might reflect the methodological limitations of immunologic assays or the postvaccination absence of antitumor responses sufficiently robust to induce disease-free or overall survival.

Multiparameter flow cytometry expands platform for assessing functional profiles and patterns of immune responses. In particular, the use of polychromatic flow cytometry is likely to assume a role in defining the correlates of protection for vaccine efficacy as well as in monitoring immunotherapies in diseases such as HIV and cancer. In fact, it allows simultaneous detection of various parameters such as enumeration at the single cell level of different T-cell subsets (naïve, effector, central memory, effector memory), as well as cells belonging to the innate and myeloid compartment and providing information about the breadth and the quality of the induced immune response. The recent literature relates simultaneous expression of multiple functions (polyfunctionality) to immunity, since measurement of IFN-*γ* alone underestimates the total response [69].

Nevertheless, despite advances in the development of immune monitoring assays during the past decade, further advances are needed to implement throughput and standardization of such assays according to Good Laboratory Practice guidelines, such as those recently formulated based on recommendations from the iSBTc-SITC/FDA/NCI Workshop on Immunotherapy Biomarkers [70].

The goal of the present paper was to provide further insights into the development of standardized FCM-based immune assays. In particular, we revised the most common and critical issues of FCM-based technologies used for immune monitoring and evaluated the applicability of a six-color flow cytometric assay, previously developed in our laboratory, for immune monitoring in the setting of melanoma studies. This assay simultaneously measures effector cell degranulation and cytokine production by Melan-A/MART-1 specific CD8^+^ T-cells. We were able to define some of the crucial aspects regarding sample processing and evaluated various staining and gating strategies. Concerning flow data analysis, we might conclude that different approaches of analysis and visual representations should be performed in order to obtain a complete picture of results about polyfunctionality of tumor specific T-cells.

## Figures and Tables

**Table 1:** 

Critical issue		References
Blood collection, shipment, and processing	Temperature of storage	[16]
	Time from blood draw to sample processing	[17, 18]
	Freezing/thawing conditions	[19]

DNAse during culture		[20]

Perm/lysing reagents		[21]

Flow cytometric issues	Antibodies and fluorochromes	[22, 23]
	Spectral overlap and color compensation Instrument setup	
	Data acquisition and analysis	[24]

**Table 2:** 

	w/o DNAse	w/o DNAse	w/o DNAse
	NT	SEB	MART-1
% of CD8^+^ MART-1^+∗^	0.0036	0.0039	0.0046
Number of CD8^+^ MART-1^+¶^	34	29	38

	DNAse in culture	DNAse in culture	DNAse in culture
	NT	SEB	MART-1

% of CD8^+^ MART-1^+^	0.013	0.0081	0.0077
Number of CD8^+^ MART-1^+^	129	80	75

	DNAse always	DNAse always	DNAse always
	NT	SEB	MART-1

% of CD8^+^ MART-1^+^	0.015	0.017	0.011
Number of CD8^+^ MART-1^+^	143	153	103

*Percentage or ^¶^number of cells as assessed in an FMT assay, performed on PBMCs from healthy donors, treated and labeled as in Figure 1, with the addition of HLA-A2/peptide tetramer staining at the beginning of culture (HLA-A2∗0201 peptide phycoerythrin (PE) tetrameric complexes specific for the Melan-A/MART-1 antigen).
